# Ecotoxicological Properties of Pure and Phosphorus-Containing Graphene Oxide Bidimensional Sheets in *Daphnia magna*

**DOI:** 10.3390/toxics12040252

**Published:** 2024-03-29

**Authors:** F. Mendoza-Villa, Noemi-Raquel Checca-Huaman, Tainara L. G. Costa, Jair C. C. Freitas, Juan A. Ramos-Guivar

**Affiliations:** 1 Grupo de Investigación de Nanotecnología Aplicada Para la Biorremediación Ambiental, Energía, Biomedicina y Agricultura (NANOTECH), Facultad de Ciencias Físicas, Universidad Nacional Mayor de San Marcos, Av. Venezuela Cdra 34 S/N, Ciudad Universitaria, Lima 15081, Peru; freddy.mendoza1@unmsm.edu.pe; 2 Centro Brasileiro de Pesquisas Físicas, Rio de Janeiro 22290-180, RJ, Brazil; nomifsc@cbpf.br; 3 Laboratory of Carbon and Ceramic Materials, Department of Physics, Federal University of Espírito Santo, Vitória 29075-910, ES, Brazil; tainara.costa@edu.ufes.br (T.L.G.C.); jairccfreitas@yahoo.com.br (J.C.C.F.)

**Keywords:** aquatic environment, biomarkers, ecotoxicity, 2D materials, graphene oxide derivatives

## Abstract

In this work, the synthesis and structural, thermal, vibrational, morphological, and electronic characterization of 2D-like pure graphene oxide (GO) and phosphorus-containing graphene oxide (GOP) sheets were investigated. The average thicknesses of GO and GOP were 0.8 μm and 3.1 μm, respectively. The electron energy-loss spectroscopy spectra were used to analyze the differences in the C-K and O-K energy edge bands between GO and GOP. In addition, colloidal stability was studied using dynamic light scattering and zeta potential physicochemical techniques, determining that as the concentration increases, the hydrodynamic diameter and electrostatic stability of GO and GOP increase. The colloidal stability was quite important to ensure the interaction between the suspended solid phase and the biomarker. The 2D-like materials were used to determine their ecotoxicological properties, such as the medium lethal concentration, a crucial parameter for understanding ecotoxicity. Acute ecotoxicity experiments (24 h) were conducted in triplicate to obtain robust statistics, with corresponding mean lethal concentration ($LC_{50}$) of 11.4 mg $L^{-1}$ and 9.8 mg $L^{-1}$ for GO and GOP, respectively. The morphological parameters of GO and GOP were compared with a negative control. However, only the case of GO was analyzed, since the *Daphnia magna* (*D. magna*) set exposed to GOP died before completing the time required for morphological analysis. The results indicate that the GOP sample is more toxic than the GO, both during and after exposure. Furthermore, the morphological parameters with the greatest statistically significant changes (p<0.05) were associated with the heart and body, while the eye and tail showed less significant changes.

## 1. Introduction

The toxicity of nanomaterials is a major contemporary concern, given the uniqueness of their properties, which are often markedly different from their macroscopic counterparts [1]. Paradigmatic examples are the plasmon effect of gold [2], the antibacterial property of silver [3], the catalytic ability of platinum [4], and the enhanced photocatalytic activity of titanium dioxide [5]. In parallel, 2D-like materials have generated significant expectations since the discovery of graphene in 2004, derived from graphite [6]. Materials such as graphene, borophene, and phosphorene, among others, exhibit properties notably superior to those known prior to their discovery [7]. This superiority is manifested in areas such as energy storage [8], electrical properties with better electrical conductivity [9], thermal properties [10], mechanical properties such as better elasticity [11], and others. The possibility of generating other types of 2D-like materials, such as graphene oxide (GO) and phosphorus-containing graphene oxide (GOP), adds even more versatility to the field. A significant distinction between GO, GOP, and graphene is that both GO and GOP can be dispersed in water [12,13], unlike graphene, which is hydrophobic. The potential for future applications of these new 2D-like materials is undeniable [14]; however, understanding and assessing their toxicity is essential to safeguarding marine biocenosis.

Environmental biomarkers, or organisms that show biological responses to changes in their environment, are used in the analysis of the toxic effects of a specific material. *D. magna* is a cladoceran a few millimeters long with a short life cycle and asexual reproduction by parthenogenesis under favorable conditions [15]. It is the most widely used environmental biomarker in toxicological tests [16]. The rapidity with which toxicity, exemplified by *D. magna* mortality, manifests as a result of GO and GOP exposure is critical. These mortalities provide valuable data for determining the $LC_{50}$. Thus, the discipline of nanotoxicology emerged, defined by Oberdörster as “the science that studies the toxic effects of nanoparticles on the environment and living beings” [17].

Although there are few studies on the toxicity of 2D-like materials [18], except for graphene and graphene oxide, which are the most widely studied [19], the scientific literature abounds on the consequences of the toxicity of nanoparticles (NPs) [20,21]. For example, titanium dioxide (TiO_2_) NPs have shown delayed reproduction in aquatic organisms [22,23], cerium dioxide (CeO_2_) NPs have generated genotoxicological damage in the DNA strands of *D. magna* and *C. riparius* [24], and phosphorus has induced both genotoxic and morphological damage in zebrafish [25].

In this work, with the aim of unraveling the ecotoxicological properties of synthesized GO and GOP, the 24 h-$LC_{50}$ was first evaluated. Moreover, the morphological analysis was conducted for 14 days after exposure to GO and GOP, providing detailed information on their significant statistical influence on the morphological parameters. The results suggest a relative significance in morphological parameters compared to the negative control, as demonstrated by the related Student’s *t*-test. The determined 24 h-$LC_{50}$ values for GO and GOP on *D.magna* will be useful, for example, as permissible concentrations for aquatic release during water remediation using the adsorption process.

## 2. Experimental Section

### 2.1. Synthesis of GO Sheets

The oxidation of graphite was carried out by a modified Hummers’ method [26]. For the synthesis, about 1 g of graphite precursor and 0.5 g of NaNO_3_ were put into 70 mL of concentrated H_2_SO_4_ (in an ice bath). Then, 3.0 g KMnO_4_ was gradually added. The mixture was stirred for 2 h and then diluted with distilled water. After that, 3.0 mL of H_2_O_2_ (35%) was added into the solution until the color of the mixture changed to brownish yellow. The obtained product was washed with distilled water until the filtrate reached a neutral pH and then lyophilized.

### 2.2. Synthesis of GOP Sheets

The GOP sheets were synthesized following a modified Tour method [27]. The amounts of H_2_SO_4_ and H_3_PO_4_ used in the reaction were changed in comparison with the route originally proposed by Tour et al. [28], while keeping the total acid volume at 140 mL, using 50 mL of H_2_SO_4_ and 90 mL of H_3_PO_4_. For the graphite oxidation reaction, about 1.0 g of the graphitic precursor was added to the H_2_SO_4_ + H_3_PO_4_ mixture, followed by the slow addition of 6.0 g of KMnO_4_. The mixture was stirred for 12 h at 50 °C and, after cooling down to room temperature, 400 mL of ice and 3.0 mL of H_2_O_2_ (35%) were added to the reaction medium. The obtained product was washed with distilled water until the filtrate reached a neutral pH and then lyophilized.

### 2.3. Characterization

X-ray diffractograms were recorded with an Empyrean diffractometer (Malvern Panalytical, Malvern, UK) with the following setup configuration: CuKα radiation at the wavelength λ = 1.54056 Å emitted by a Cu anode operating at 45 kV and 40 mA. X-ray diffraction (XRD) data were obtained for the 2θ = ${\mathrm{5–35}}^{\circ }$ range using Bragg–Brentano geometry in spinner mode with an angular step of $0.026^{\circ }$.

Thermogravimetry (TG) measurements were conducted using Shimadzu equipment (Kyoto, Japan) in the temperature range from 27 to 900 ^∘^C. The samples were analyzed under a synthetic air atmosphere (50 mL $\min^{-1}$ flow rate) and a heating rate of 10 ^∘^C/min.

The RT infrared (IR) spectra were measured using an IRPrestige-21 Shimadzu spectrophotometer. The analyzed IR frequency range was in the wavenumber interval from 400 to 4000 ${\mathrm{cm}}^{-1}$, with an optical resolution of 2 ${\mathrm{cm}}^{-1}$.

The C13 NMR experiments were conducted at room temperature in a Varian-Agilent spectrometer operating at 100.5 MHz (9.4 T magnetic field). Magic angle spinning (MAS) at 14 kHz was employed in all experiments, with the powdered samples packed into 4 mm diameter zirconia rotors. The pulse sequence was composed of a π/2 pulse (4.3 μs) immediately followed by a pair of π pulses (8.6 μs) and the subsequent detection of the free induction decay (FID), followed by a recycle delay of 15 s. The spectra were obtained by Fourier transform of the FIDs, after accumulation of ca. 4000 transients, and the chemical shifts were referenced to tetramethylsilane (TMS), using hexamethylbenzene as a secondary reference.

The morphology, size, and elemental content of the powders were determined using a TESCAN LYRA3 high-resolution scanning electron microscope (SEM) working with a FEG-type electron source and an Oxford Energy Dispersive Spectroscopy (EDS) detector (Tescan Brno s.r.o., Brno, Czech Republic). Secondary electron imaging and atomic element mapping were captured with a 5 kV accelerating voltage and a 9 mm working distance.

Morphological and size evaluation was also conducted by electron imaging microscopy (EM) with two modes: transmission (TEM) and high-resolution (HRTEM), employing a 200 kV JEOL 2100F instrument (Tokyo, Japan). Further, the elemental compositions were investigated by electron energy-loss spectroscopy (EELS). EELS measurements were obtained in the scanning TEM imaging mode considering a 0.7 nm spot size, 5 mm spectrometer aperture, and 1.8 eV energy resolution. The optimized dispersions were used in small liquid volumes for the measurements, and they were dried on the microscope equipment’s sample holder.

### 2.4. D. magna Culture

The cladoceran *D. magna* was cultured under normal optimal conditions where it did not generate damage or alterations. The unfavorable culture conditions may cause individual deaths or affect asexual reproduction and birth, which is of utmost importance in ecotoxicity experiments since they require female daphnia (parthenogenesis cycle), not males, the last being easily differentiated by featured morphologies [29]. Specifically, the standardized optimal condition parameters involved are: First, the circadian rhythm was taken into account, and then the individuals were subjected to an 8:16 h light:dark photoperiod with a pH of 7.5 ± 0.5, and a temperature of (20 ± 1) ^∘^C. Second, the beakers used for the culture populations had a total volume of 250 mL, but only 200 mL were used for culture purposes to avoid spills and facilitate daphnia mobility among individuals. Third, the individuals were fed with a mixture of microalgae of the genus *Chlorella Vulgaris* during the culture time (not fed during exposure), with a corresponding scale of 1 mL per 100 mL of volume (total volume of 200 mL). Finally, the exoskeleton molts were removed daily, and the individuals were transferred to new beakers every three days. This process was continued until the hatchlings of the second generation were reared under the optimal conditions established in the laboratory. A total of 70 individuals was obtained. From them, 310 neonates were born and were used for exposure experiments. A total of 10 neonates were used for negative control (N.C.).

### 2.5. D. magna Exposure to GO and GOP Sheets

Unlike graphene, which has long been considered a hydrophobic surface [30], GO is considered a hydrophilic surface [31]. However, before performing the ecotoxicity experiment, a protocol to correctly disperse the two GO derivatives was performed. The results regarding the dispersion of both samples using the sonication method (BIOBASE sonicator) were applied in a volume of 200 mL for three types of water.

- **Commercial drinking water:** The GO could not be properly dispersed in this type of water, as the GO particles were totally flocculated.
- **Ultrapure water:** In the ultrapure water, both samples could be properly dispersed. This suspension was evidenced in other previous works regarding the dispersion of GO [32].
- **Microalgae water:** In the microalgae water, as in the case of the commercial drinking water, the GO could not be dispersed either. The substantial difference with the commercial drinking water was that it took a little longer for the GO particles to be suspended.

This experiment demonstrates the property of both samples being able to disperse in an adequate dispersive medium [12,32]. On the other hand, it has been observed that GO can be easily dispersed at a neutral pH [13,31]. This dispersion in water is possible due to the hydrophilic chemical groups that stabilize the GO and GOP electrostatically in water [31,33]. Figure S1a shows the experiments to disperse the GO after a time of 24 h. To disperse the GOP, a sonicator was used, with which, after a sonication of 15 min, a concentration of 1500 mg $L^{-1}$ of GOP could be dispersed. For both GO and GOP, dispersion of these 2D-like materials was observed at very high concentrations. Therefore, the concentrations for the ecotoxicity experiment were made by means of a stock concentration and then diluted until the desired concentration was obtained, as shown in Figure S1b. Already with the mother concentrations and with the set of *D. magna* belonging to the second generation of the culture, ecotoxicity experiments were performed for different concentrations of GO and GOP with a population of 10 neonates per replicate (×3) in a beaker with 200 mL of ultrapure water.

### 2.6. Morphological Analysis of D. magna and Statistical Analysis

The morphological analysis of *D. magna* was carried out after 14 days of exposure to GO and GOP, with the objective of observing whether there were significant morphological changes or malformations in adult individuals. For this purpose, a Greetmed microscope model DN117M was used to measure morphological parameters, such as tail, body, antenna, heart, and eye, using ScopeImage 9.0 software. The box plots were used to represent the distribution of the set of data obtained for the morphological parameters. For example, for N.C. (10 neonates), the body had values of 2222, 2084, 2367, 2330, 2138, 2152, 2004, 2148, 2299, and 2056 μm. In addition, for survival neonates exposed to 5 mg $L^{-1}$ of GO (7 neonates), the body reported values of 2188, 1800, 2067, 1786, 2023, 1902, and 1745 μm. The same was performed for all of the other concentrations and morphological parameters. The reason why the error bars overlapped with the boxes is that the distribution is centered around the mean value of the data set. The vertical lines are also named whiskers in pure statistical theory, and their length is related to the minimum and maximum values of the data set. The OriginPro 9.0 software was used to obtain the box plots.

Subsequently, it was verified whether the data followed a normal distribution, for which the Shapiro–Wilk normality test was used. After this verification, a Student’s *t*-test was performed to determine the significance of the morphological parameters using SPSS statistical software v.27. The criterion for significance was a *p*-value equal to or less than 0.05.

## 3. Results and Discussions

### 3.1. Structural and Thermogravimetric Analyses

**Figure 1:**
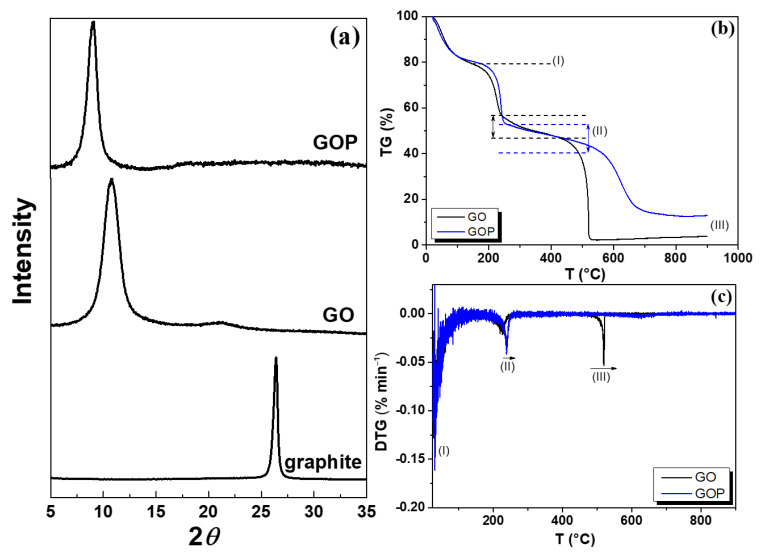
(**a**) X-ray diffractograms for GOP, GO, and graphite. (**b**) TG and (**c**) DTG for GO and GOP samples.

Figure 1a shows the X-ray diffractograms and the characteristic Bragg peak at $26.4^{\circ }$ (0.337 nm-d spacing) with the (002) Miller index for crystalline graphite precursor [34]. On the other hand, the GO sheets showed two diffraction peaks at $10.8^{\circ }$ and $21.3^{\circ }$. The GOP sheets only revealed a diffraction peak at $9.1^{\circ }$. The onset of oxygen-containing functional groups between the graphite layers and the intercalation of water molecules are responsible for the increase in *d*, the interlayer spacing, to 0.8185 nm and 0.9710 nm for GO and GOP, respectively [35]. It is worth mentioning that reduction in GO did not occur in our samples because the common broad peak with 0.36 nm of interlayer spacing was not observed [36].

Thermograms in Figure 1b showed three marked regions for weight loss. Region (I), related to 10% of the loss, is related to hydroxyl, epoxy, and physiosorbed water from 27 to 200 ^∘^C. Region (II) from 200 to 450 ^∘^C assigned to between 45 and 50% of the loss weight for GO and GOP indicates the release of the oxygen-containing groups [35]. While Region (III) occurs immediately after reaching 450 ^∘^C, it was observed as an abrupt decay related to the almost total carbon burning decomposition in both GO structures [35]. The derivative thermogram (DTG), as shown in Figure 1c, revealed two peaks for both phases. They occur at 220 ^∘^C and 239 ^∘^C for the GO and 521 and 627 ^∘^C for the GOP. It seems that the phosphorus-containing GO system is thermally more stable than a single GO, maybe due to the presence of the remaining phosphorus chemical groups.

### 3.2. Vibrational Analysis

**Figure 2:**
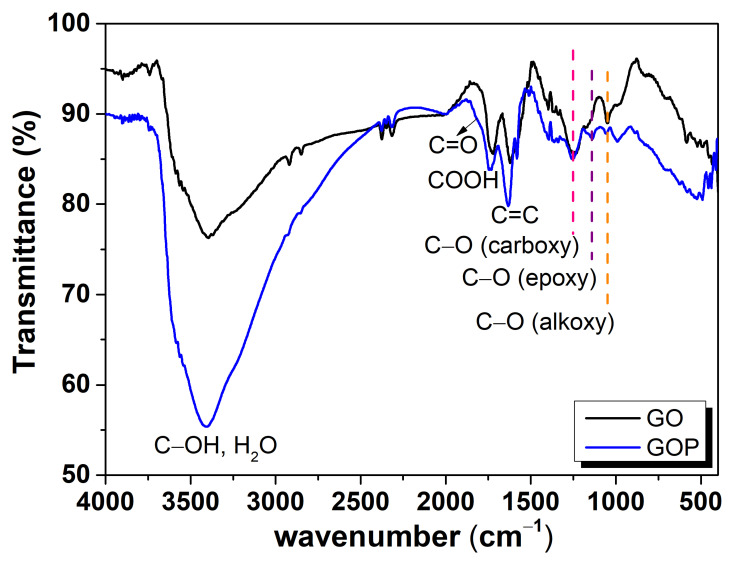
IR vibrational spectrum for GO and GOP samples. Vertical dash lines indicate the carboxy, epoxy, and alkoxy functional groups found in the samples.

IR optical active groups for GO were confirmed by FTIR analysis. Figure 2 shows the IR spectra for both samples. C−O stretching vibrations for alkoxy, epoxy, and carboxyl groups were found at 1053, 1177, and 1276 ${\mathrm{cm}}^{-1}$ [37], respectively. The C=C featured IR band was found in the region from 1624 to 1636 ${\mathrm{cm}}^{-1}$ for GO and GOP. An intense carbonyl group, ν(C=O) in COOH, and a weak C=O stretching ketonic group were observed in 1737 and 1809 ${\mathrm{cm}}^{-1}$. Hydroxyl stretching modes of organic groups (C-OH) and water were found near 3400 ${\mathrm{cm}}^{-1}$.

### 3.3. NMR Analysis

**Figure 3:**
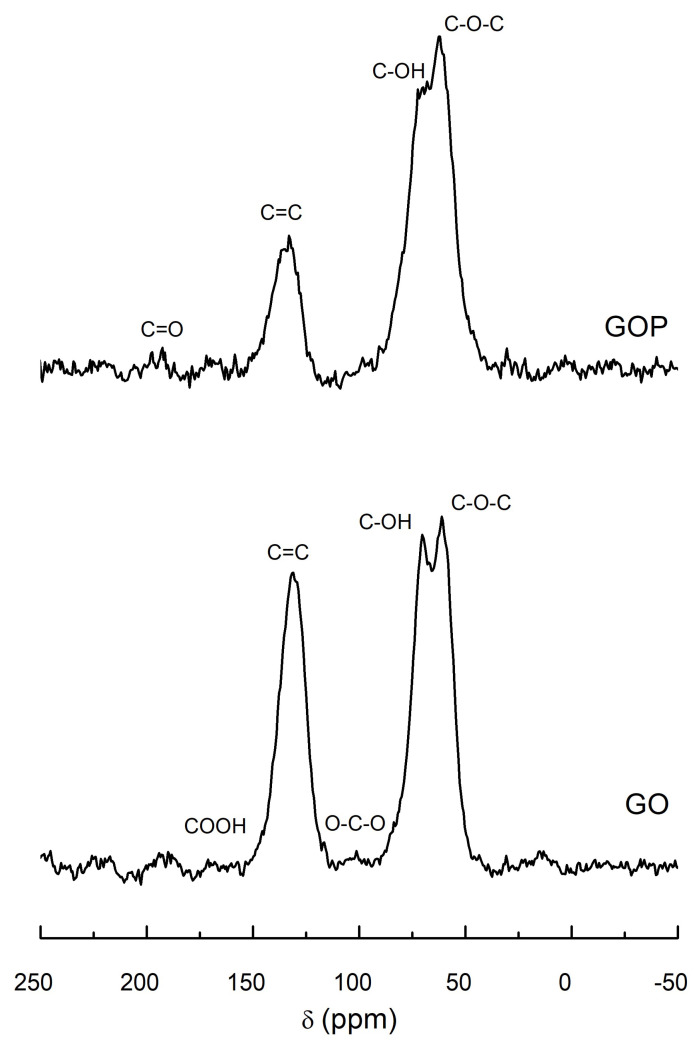
C13 NMR spectra of GO (**bottom**) and GOP (**up**) samples.

The C13 NMR spectra of GO and GOP samples are shown in Figure 3. Both spectra exhibit resonances indicative of oxygen-containing functional groups characteristic of the structure of graphene oxides, with prominent peaks corresponding to epoxy (62 ppm) and hydroxyl (70 ppm) functionalities. Additionally, less abundant and weaker signals corresponding to other oxygenated functional groups, such as lactol (101 ppm), carbonyl (192 ppm), and carboxyl (167 ppm) groups, are also detected. The other dominant resonance in these spectra occurs at a chemical shift around 132 ppm, corresponding to C13 nuclei in hexagonal rings formed by ${\mathrm{sp}}^{2}$-hybridized carbon atoms, which are a common feature of both graphene oxides and graphite oxides [28,38]. The relative intensities of the resonances due to the oxygen-containing functional groups (mainly epoxy and hydroxyl groups) are higher in the case of the GOP sample, which agrees with previous reports indicating the high oxidation degree of samples prepared by the Tour method and its variations [27,28,39].

### 3.4. Colloidal Stability Analysis

**Figure 4:**
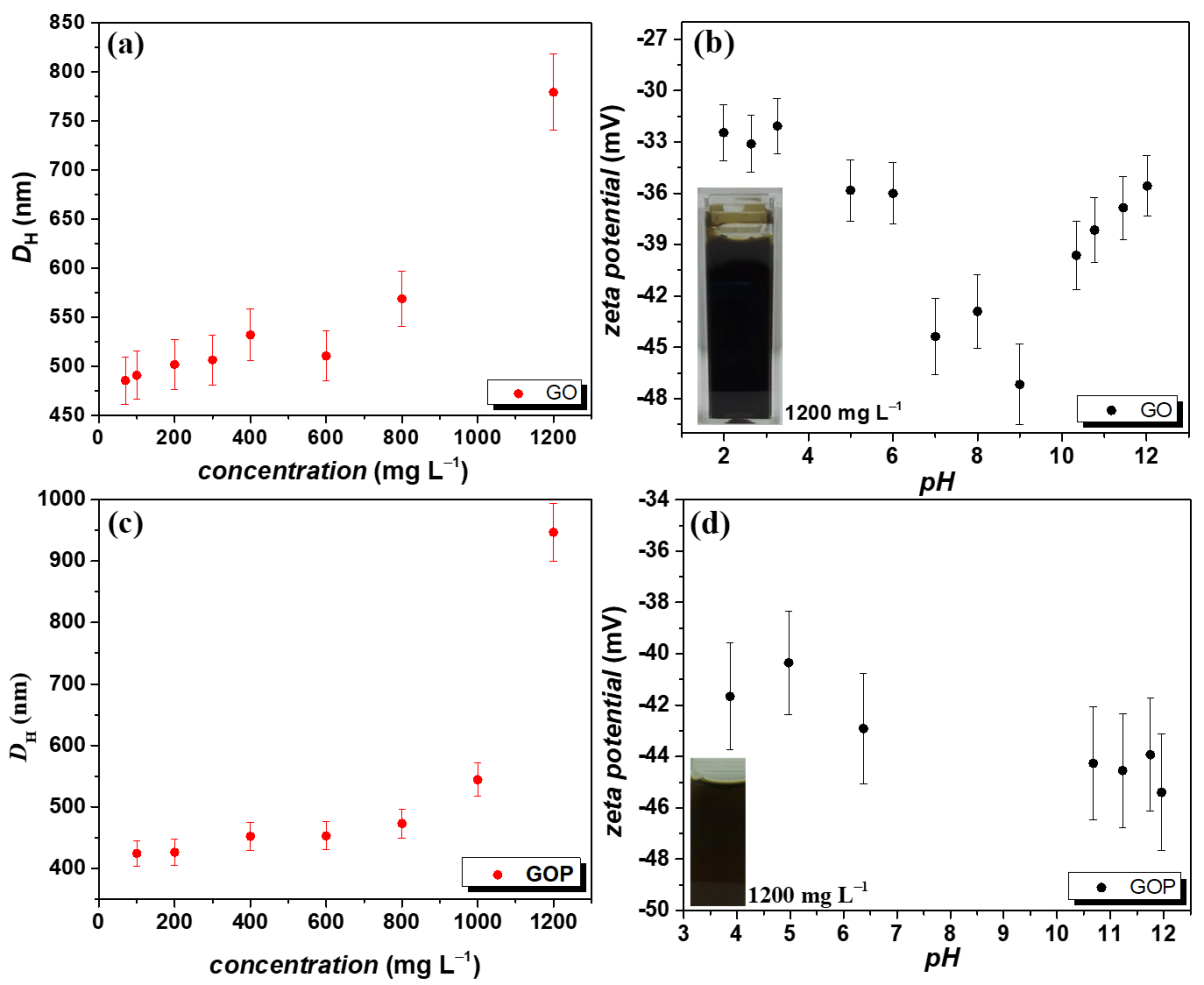
(**a**–**c**) $D_{H}$ vs. concentration plot. (**b**–**d**) pH dependence of zeta potential for GO and GOP samples. The inset in (**b**,**d**) represents the initial suspension color for colloidal stability measurements.

The effective diameter against the pH for GO and GOP suspensions at pH = 7 is shown in Figure 4a,c. When increasing the concentration, the effective diameter increases due to aggregate formation. The zeta potential measurements for both samples, as given in Figure 4b,d, indicate high colloidal stability above −30 mV in the whole pH range. At neutral pH, the zeta potential reached values of ca. −42 mV.

### 3.5. SEM and TEM Analysis

**Figure 5:**
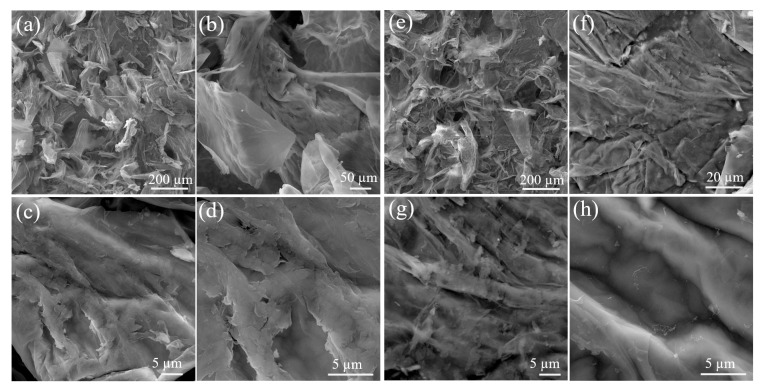
(**a**–**d**) SEM images at various magnifications for GO and (**e**–**h**) GOP sheets.

Figure 5a–h show the SEM images for the 2D-like structure sheets of GO and GOP at various magnifications. It was observed to have a polycrystalline behavior with diverse microscopic lengths. P containing GO has been confirmed in contrast to the GO sample. The EDS is given in Figure S2.

**Figure 6:**
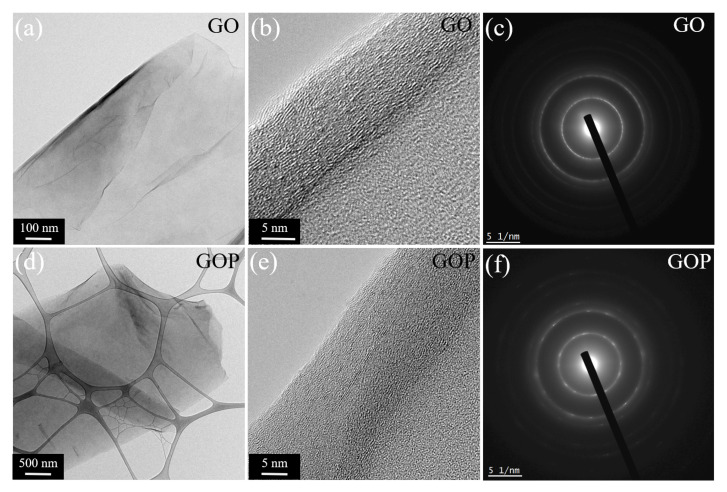
TEM images and SAED patterns for (**a**–**c**) GO and (**d**–**f**) GOP samples.

Figure 6a–f confirmed the bidimensional morphologies, where the mean thickness was 0.8 μm and 3.1 μm for the GO and GOP samples, respectively.

**Figure 7:**
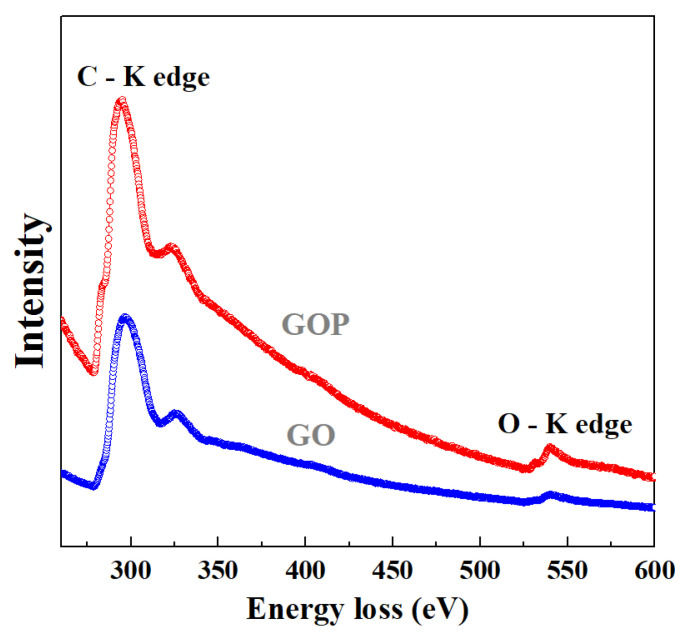
C−K and O−K edges in the EELS spectra for GO and GOP samples.

The C-K and O-K edges were analyzed, and they are shown in Figure 7. In the case of GO, the two energy bands were found for the C−K region [40]: (i) a small shoulder at 284.8 eV (1s-$\pi^{*}$ electronic transition for C=C bonds) and (ii) a strong band at 293.8 eV (1s-$\sigma^{*}$ electronic transition for C). The O−K edge revealed the presence of two marked regions at 531.6 eV (retained adsorbed water or epoxy groups) and 539.9 eV (hydroxyl groups) [41]. For the GOP, the C-K region showed only one energy band at 296.3 eV, and, for the O-K edge, only the band at 540.3 eV was found. It was also observed that the first band for the C−K edge is assigned to the C−O bonding [42]. Hence, the lack of this bond in the GOP sample can be related to structural defects in the GO framework. Consequently, both samples exhibited characteristic energetic contributions to GO.

### 3.6. Lethal Concentration (24 h-$LC_{50}$)

**Figure 8:**
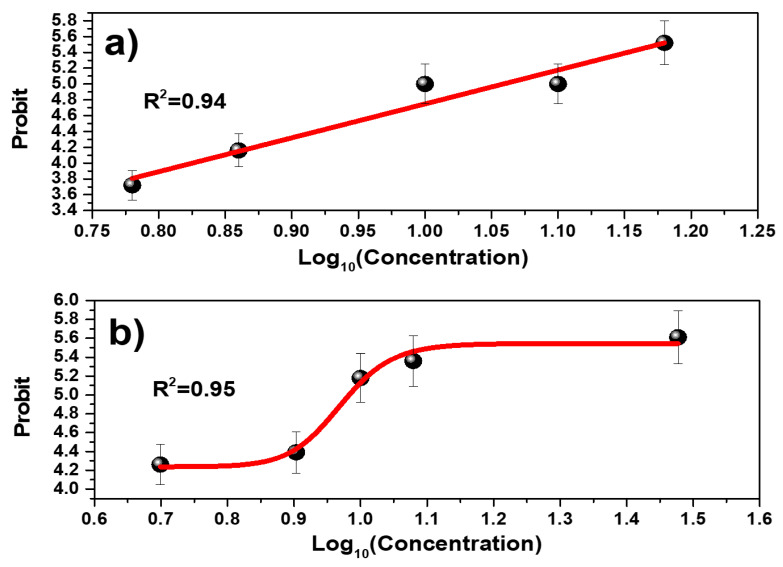
Probit plot for (**a**) GO and (**b**) GOP sheets.

The mortality results shown for GO have a linear trend, while for GOP they have a logistic trend as shown in Figure 8. As can be seen, the $R^{2}$ of the mortality curve is very close to the value of one. This demonstrates that the fit adequately describes the data. Equations (1) and (2) model the mortality, and they have the following corresponding form:

- **For GO sample:**
  (1) Y=4.28·X+0.47
- **For GOP sample:**
  (2) $$Y=5.54-1.3/(1+{\left(1.03\cdot X\right)}^{25})$$

In the Probit method, the variable *Y* represents mortality in Probit units, while the variable *X* represents the logarithm in base 10 of the concentration [43]. The reason why the Probit method is used instead of simply plotting mortality in percentages is because it allows the $LC_{50}$ value to be calculated by a mortality fit using probability techniques and also estimates values that follow a lognormal tolerance distribution [44]. However, the results can also be displayed in percent vs. concentration or in percent vs. logarithm of concentration [45].

To find the $LC_{50}$ value, it is necessary to replace Y=5.0 in the equations found by means of regression. When performing this calculation, it is obtained that the $LC_{50}$ of GO is equal to 11.4 mg $L^{-1}$ while the $LC_{50}$ of GOP is equal to 9.8 mg $L^{-1}$. This suggests that GOP is much more toxic than GO when exposed to the environmental biomarker *D. magna* at 24 h acute toxicity. Thus, environmental biomarkers are important for evaluating the materials’ toxicities, allowing for the determination of lethal concentrations for aqueous applications [46].

It was also seen that the Probit curve has a different trend behavior; while, for GO, the extreme values are relatively close to its $LC_{50}$ value, for GOP, this does not occur since the extreme values could not be calculated accurately. The mortality curves shown in Figure 8 are the expected ones since many other works show this type of trend when the regression curve is obtained following the Probit method, which is one of the most used when performing toxicological tests.

When comparing the $LC_{50}$ value of this 2D-like material with others applied to *D. magna*, a notorious difference was observed. For example, for commercial nanomaterials such as TiO_2_ NPs and TiO_2_ NWs, the $LC_{50}$ ranges from 118 mg $L^{-1}$ to 224 mg $L^{-1}$ [23,47]. In the case of nanomaterials including different compounds, such as γ-Fe_2_O_3_-TiO_2_-GO, the $LC_{50}$ value is at 550 mg $L^{-1}$ [48]. However, when considering only GO sheets, $LC_{50}$ values as low as 0.18 mg $L^{-1}$ have been recorded in studies with 24 h of exposure [49]. On the other hand, $LC_{50}$ values of 45.4 mg $L^{-1}$ and 84.2 mg $L^{-1}$ have been found in different investigations, but in this case, the GO dimensions and exposure time vary [32,50]. In another investigation, the $LC_{50}$ value of GO functionalized with nanomaghemite is too toxic, its $LC_{50}$ being 0.94 mg $L^{-1}$, while, for multiwall carbon nanotubes functionalized with nanomaghemite, its $LC_{50}$ was 381.8 mg $L^{-1}$ [51]. GO also shows toxicity in other aquatic organisms, such as zebrafish, where delayed egg hatching was observed, as well as morphological damage, even at relatively low concentrations [52]. Table 1 largely summarizes the $LC_{50}$ of GO-related materials in the environmental biomarker *D. magna*.

As seen in Table 1, the $LC_{50}$ is highly variable since, although all are exposed to GO, the exposure time gives us very different $LC_{50}$ values. In addition, functionalizing GO with another material can both increase or decrease the $LC_{50}$ value. Therefore, it is imperative to conduct further experiments in order to obtain a more complete understanding of the ecotoxicity caused by this 2D-like material.

This confirms that both 2D-like materials, GO and GOP, are toxic. However, if they are functionalized with some other material their $LC_{50}$ may vary. As shown, when it is functionalized with γ-Fe_2_O_3_–TiO_2_, its value increased [48], while, when it is doped with nanomaghemite, its value decreased [51]. Furthermore, it was observed that the 2D-like GOP material is much more toxic than the 2D-like GO material, as in the days after exposure, the surviving *D. magna* assemblage started to die on the second day of the control, culminating in death at the latest on the fourth day of the control. For the same reason, only the morphological analysis of the *D. magna* set was performed for GO, as they were able to survive the 14 days after exposure to GO. This analysis was performed using the Student’s *t*-test, which is a useful test to determine significance [53].

Phosphorus toxicity has been studied in other aquatic organisms, such as zebrafish, which is a vertebrate environmental bioindicator [54]. The type of phosphorus used is black phosphorus, which is also a 2D-like material. The effects produced on zebrafish are both genotoxicological and morphological [25]. It is also mentioned that black phosphorus affects locomotor development and causes DNA damage in zebrafish larvae [55]. Therefore, if a much larger biomarker such as zebrafish is adversely affected by phosphorus, then it is possible that phosphate groups containing GO could lead to mortality and malformations in *D. magna* when GOP is applied.

### 3.7. Morphological Analysis in D. magna

**Figure 9:**
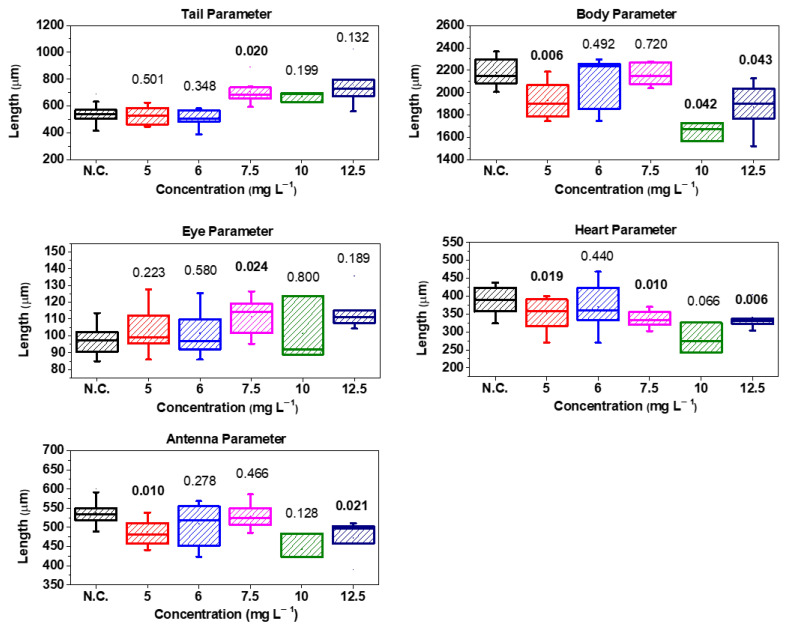
Box plot of all morphological parameters (tail, body, heart, antenna, and eye) measured after GO exposure. Numbers above the boxes are their *p*-values, and those written in bold indicate significance (*p*-value < 0.05).

Figure 9 shows these results using the box plot [56], similar to previous reports on ecotoxicity in *D. magna* [23,48,51].

The morphological parameters studied are important in the survival, reproduction rate, and behavior of young and adult *D. magna* individuals. The antenna parameter is important because it allows the locomotion of *D. magna* in the aquatic environment. Figure 9 shows only a significant change in the first and last concentrations. The body parameter is the parameter that allows the buoyancy of *D. magna* in the aquatic environment. Figure 9 showed this parameter was one of those with the greatest significant change, so it can be mentioned that the GO alters the body dimensions of *D. magna*. The eye and tail parameters were the least significant and were only present at a concentration of 7.5 mg $L^{-1}$, so it can be said that exposure to GO does not affect the morphological parameters of *D. magna* in the medium term. The heart parameter, as well as the body parameter, is significant in three of the five applied concentrations, which means that a level of stress occurs even days after exposure to GO. It can be summarized that the most significant morphological change was in the body and the heart, while the least significant morphological change occurred in the tail and the eye.

**Figure 10:**
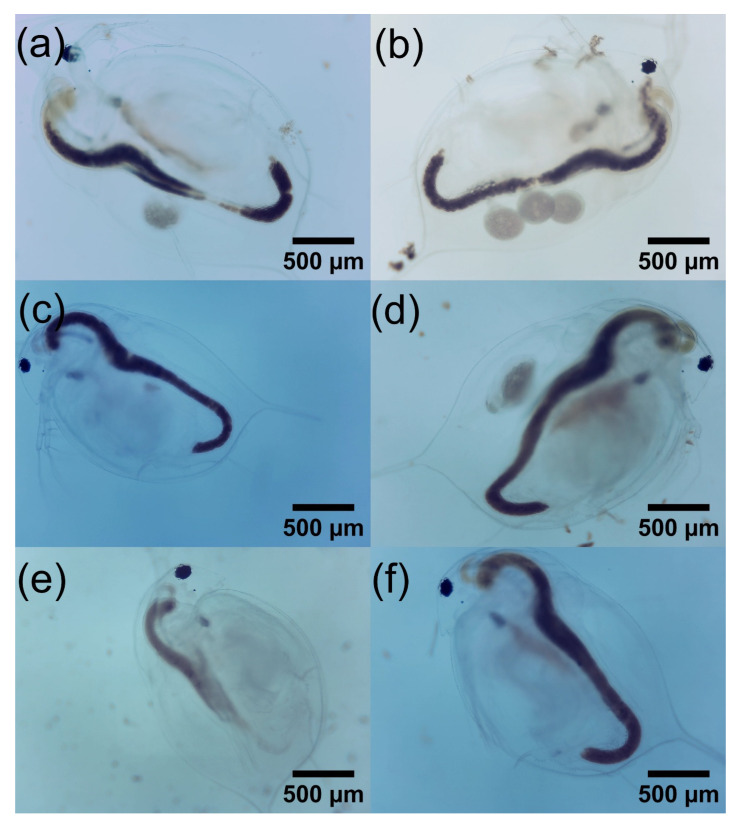
Images of *D. magna* on its last day of control. (**a**) N.C., (**b**) 5 mg $L^{-1}$ GO, (**c**) 6 mg $L^{-1}$ GO, (**d**) 7.5 mg $L^{-1}$ GO, (**e**) 10 mg $L^{-1}$, and (**f**) 12.5 mg $L^{-1}$ GO.

**Figure 11:**
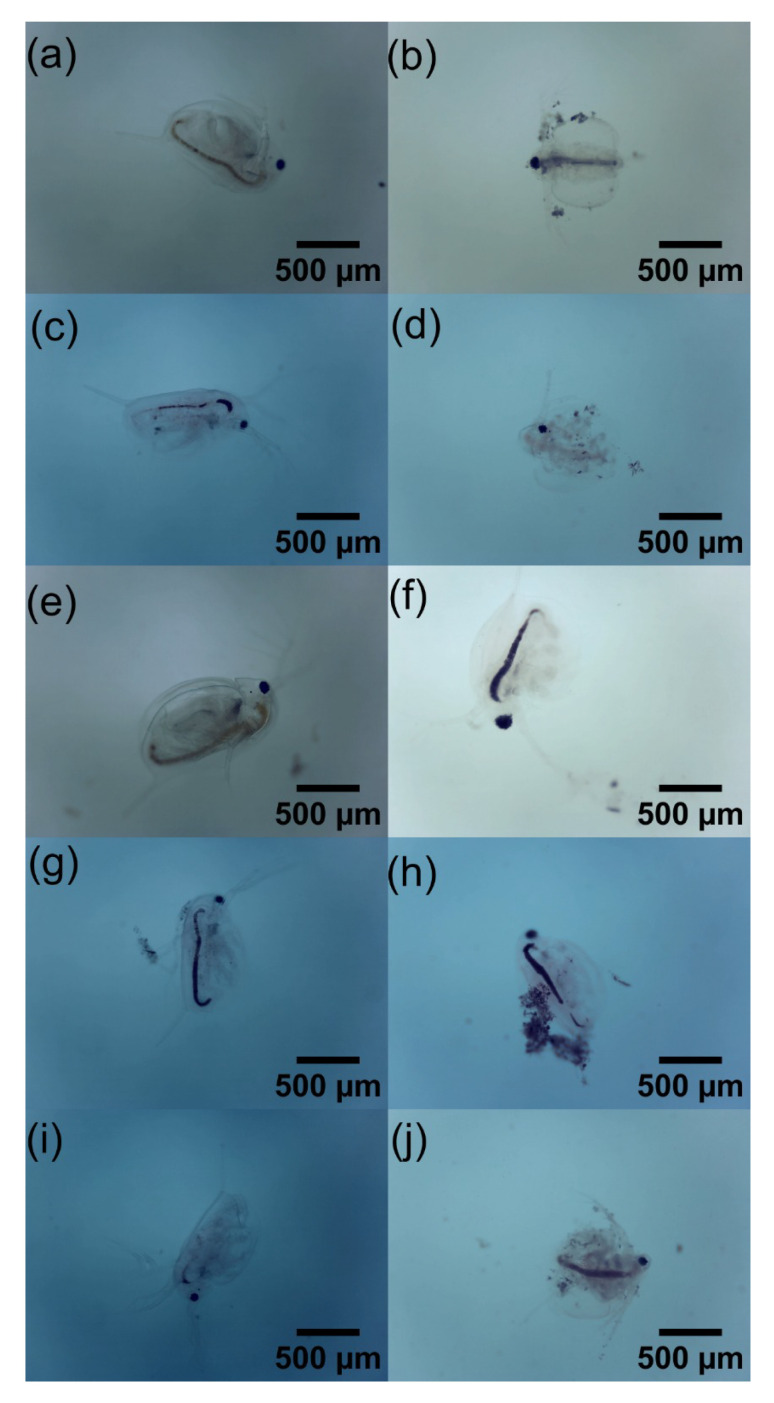
Micrographs for the GOP, (**a**) *D. magna* live at 5 mg $L^{-1}$, (**b**) *D. magna* dead at 5 mg $L^{-1}$, (**c**) *D. magna* live at 8 mg $L^{-1}$, (**d**) *D. magna* dead at 8 mg $L^{-1}$, (**e**) *D. magna* live at 10 mg $L^{-1}$, (**f**) *D. magna* dead at 10 mg $L^{-1}$, (**g**) *D. magna* live at 12 mg $L^{-1}$, (**h**) *D. magna* dead at 12 mg $L^{-1}$, (**i**) *D. magna* live at 30 mg $L^{-1}$, and (**j**) *D. magna* dead at 30 mg $L^{-1}$.

On the other hand, the concentrations at which there is greater significance are 5 mg $L^{-1}$, 7.5 mg $L^{-1}$, and 12.5 mg $L^{-1}$, while the concentration at which there was less significance was 6 mg $L^{-1}$. Regarding the reproduction of *D. magna*, the birth of individuals was observed during the period of the experiment. While the set of *D. magna* exposed to concentrations of GO of 6 mg $L^{-1}$, 7.5 mg $L^{-1}$, and 12.5 mg $L^{-1}$ also exhibited births, the number of individuals was lower than in the N.C. group. All of this is for the second repetition of GO concentrations. On the contrary, for the GOP, no birth of individuals was observed since, as shown by the very high mortality in the days following exposure, these daphnia could not reach reproduction, as was the case with GO and the N.C. Finally, Figure 10 shows images of *D. magna* on the last day of control with GO, and Figure 11 shows a comparison between live and dead *D. magna* due to the GOP.

## 4. Conclusions

2D-like sheet structures were successfully synthesized using Hummers’ method and the Tour method. The thickness was determined from TEM images, which depicted mean values of 0.8 µm and 3.1 μm for GO and GOP, respectively. The GO functional groups were confirmed by diverse physicochemical analyses. The suspension preparation method using sonication was important for monitoring the colloidal stability of GO suspensions and thus direct interactions with *D. magna*. The ecotoxicity of 2D-like materials derived from GO and phosphorus-modified GO, applied to the environmental biomarker *D. magna*, proved to be relatively toxic compared to other nanomaterials used in ecotoxicity experiments. It is worth noting the importance of specifically addressing phosphorus toxicity in GOP, which precluded the comprehensive morphological study of *D. magna* compared to GO, for which a morphological analysis was performed using a Student’s t-test to assess significance compared to a negative control. Finally, one of the highlights of this work was obtaining a $LC_{50}$ value for both 2D-like materials: 11.4 mg $L^{-1}$ and 9.8 mg $L^{-1}$ for GO and GOP, respectively. This is especially relevant for the GOP, since previously no information on its corresponding $LC_{50}$ was available, and the $LC_{50}$ values for GO vary depending on the GO size.

## Figures and Tables

**Table 1:** Ecotoxicological parameters for GO-derived materials found in the literature.

Species	System	Dispersion Method	Size	Exposition Time (h)	${\mathrm{LC}}_{50}$ (mg $L^{-1}$)	Reference
*D. magna*	GO	Sonication	0.2–0.3 μm	72	45.4	[32]
*D. magna*	GO	Sonication	0.5–5 μm	48	84.2	[50]
*D. magna*	GO-γ-Fe_2_O_3_	Sonication	10.4 nm	24	0.94	[51]
*D. magna*	γ-Fe_2_O_3_-TiO_2_-GO	Sonication	19.6 nm	24	550	[48]
*D. magna*	GO	Sonication	0.5–5 μm	72	111.4	[50]
*D. magna*	GO	Sonication	3–4 μm	24	0.18	[49]
*D. magna*	GOP	Sonication	3.1 μm	24	9.8	This work
*D. magna*	GO	Sonication	0.8 μm	24	11.4	This work

## Data Availability

The original data related to this research can be asked for any time upon request to the corresponding author’s email: juan.ramos5@unmsm.edu.pe.

## Supplementary Materials

The following supporting information can be downloaded at https://www.mdpi.com/article/10.3390/toxics12040252/s1, Figure S1: (a) Dispersion of GO in different types of water (left: microalgae water, middle: commercial drinking water, right: ultrapure water), (b) Dispersion at a concentration of 1500 mg $L^{-1}$ of GOP (orange label) and GO (green label); Figure S2: EDS spectrum for GO and GOP samples.
