# Supplementary material for: Ecotoxicological Properties of Pure and Phosphorus-Containing Graphene Oxide Bidimensional Sheets in Daphnia magna
Source: Toxics. 2024 Mar 29;12(4):252. doi: 10.3390/toxics12040252 (PMC11054868; doi:10.3390/toxics12040252)
Supplement: Supplementary file 1 [file toxics-12-00252-s001.zip › main.pdf]

toxics

Supplementary Material

Supplementary Material for Ecotoxicological Properties of Pure and Phosphorus-Containing Graphene Oxide Bidimensional Sheets in *Daphnia magna*

F. Mendoza-Villa [1], Noemi-Raquel Checca-Huaman [2], Tainara L. G. Costa [3], Jair C. C. Freitas [3] and Juan A. Ramos-Guivar [1,*]

[1] Grupo de Investigación de Nanotecnología Aplicada Para la Biorremediación Ambiental, Energía, Biomedicina y Agricultura (NANOTECH), Facultad de Ciencias Físicas, Universidad Nacional Mayor de San Marcos, Av. Venezuela Cdra 34 S/N, Ciudad Universitaria, Lima 15081, Peru; freddy.mendoza1@unmsm.edu.pe

[2] Centro Brasileiro de Pesquisas Físicas, Rio de Janeiro 22290-180, RJ, Brazil; nomifsc@cbpf.br

[3] Laboratory of Carbon and Ceramic Materials, Department of Physics, Federal University of Espírito Santo, Vitória 29075-910, ES, Brazil; tainara.costa@edu.ufes.br (T.L.G.C.); jairccfreitas@yahoo.com.br (J.C.C.F.)

Correspondence: juan.ramos5@unmsm.edu.pe

Supplementary Figures

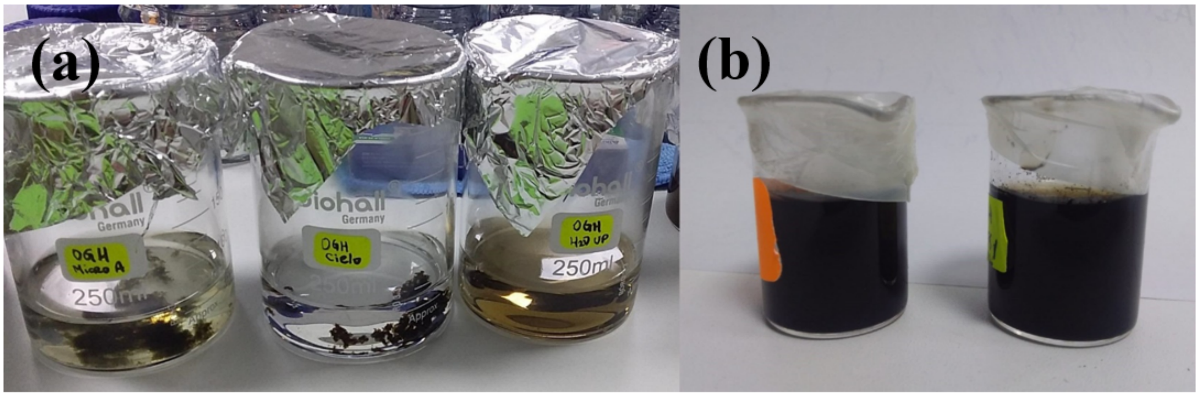

Figure S1: (a) Dispersion of GO in different types of water (left: microalgae water, middle: commercial drinking water, right: ultrapure water), (b) Dispersion at a concentration of 1500 mg L^{-1} of GOP (orange label) and GO (green label).

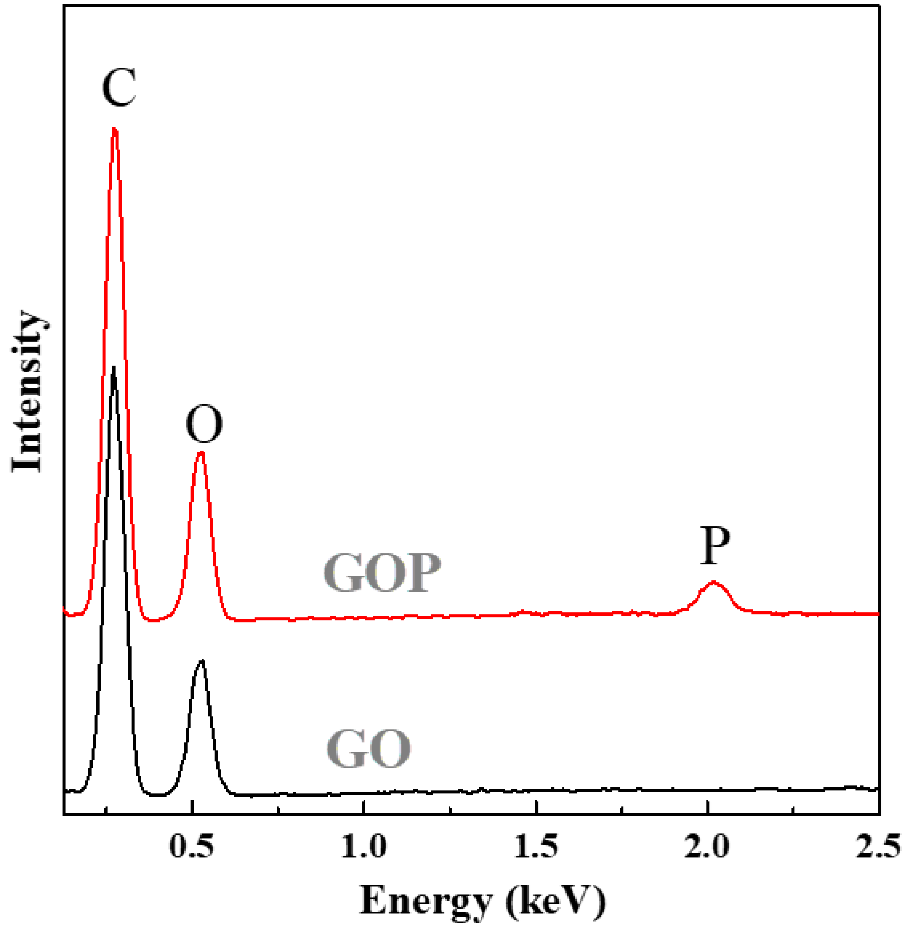

Figure S2: EDS spectrum for GO and GOP samples.
