# Supplementary figures and images for: Ecotoxicological Properties of Pure and Phosphorus-Containing Graphene Oxide Bidimensional Sheets in Daphnia magna
Source: Toxics. 2024 Mar 29;12(4):252. doi: 10.3390/toxics12040252 (PMC11054868; doi:10.3390/toxics12040252)

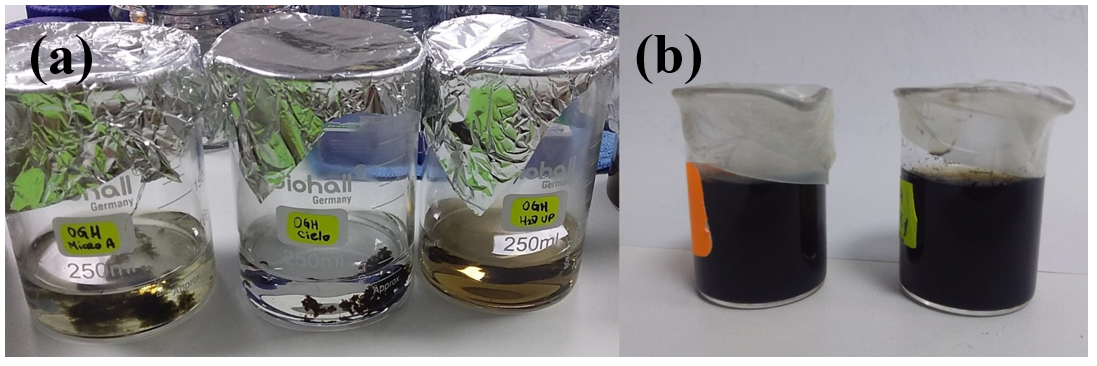

Supplement: Supplementary file 1 [file toxics-12-00252-s001.zip › FiguraS1.png]

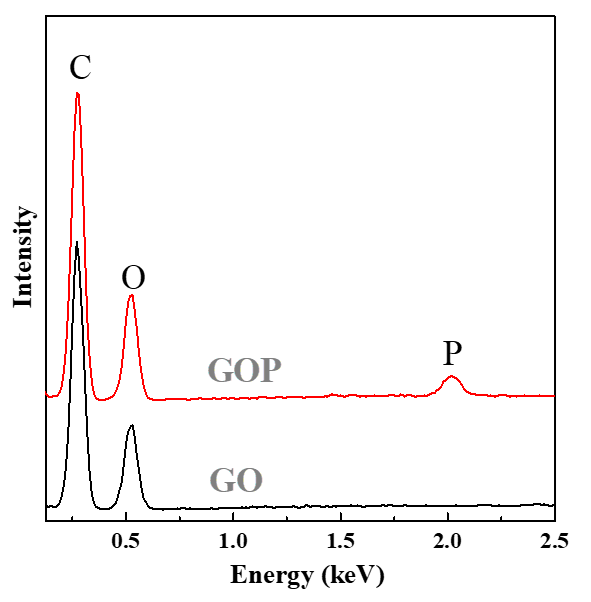

Supplement: Supplementary file 1 [file toxics-12-00252-s001.zip › FiguraS2.png]

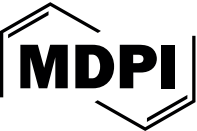

Supplement: Supplementary file 1 [file toxics-12-00252-s001.zip › logo-mdpi-eps-converted-to.pdf]

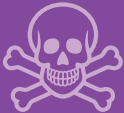

*toxics*

Supplement: Supplementary file 1 [file toxics-12-00252-s001.zip › toxics-logo-eps-converted-to.pdf]
